# 基于蛋白质沉淀的药物靶点筛选方法的研究进展

**DOI:** 10.3724/SP.J.1123.2023.11019

**Published:** 2024-07-08

**Authors:** Tong LIU, Weijie QIN, Hongjun YANG

**Affiliations:** 1.中国中医科学院医学实验中心,中医药防治重大疾病基础研究北京市重点实验室,北京 100700; 1. Experimental Research Center, China Academy of Chinese Medical Sciences, Beijing Key Laboratory of Traditional Chinese Medicine Basic Research on Prevention and Treatment for Major Diseases, Beijing 100700, China; 2.医学蛋白质组全国重点实验室,国家蛋白质科学中心(北京),北京蛋白质组研究中心, 军事科学院军事医学研究院,北京 102206; 2. State Key Laboratory of Medical Proteomics, National Center for Protein Sciences (Beijing), Beijing Proteome Research Center, Beijing Institute of Lifeomics, Beijing 102206, China

**Keywords:** 生物质谱, 药靶鉴定, 蛋白质沉淀, 蛋白质组学, biomass spectrometry, drug target identification, protein precipitation, proteomics

## Abstract

药物靶点是体内与药物分子结合的生物大分子。因此药物靶点的系统鉴定对于充分认识药物作用机制、疗效以及可能的副作用至关重要;同时全面的靶点识别可以加速药物发现及候选药物筛选的进程。生物质谱具有采集速度快、分辨率和灵敏度高等特点,是蛋白质组深度鉴定和准确定量的关键工具;基于质谱的蛋白质组学技术有助于系统性鉴定与小分子药物结合的蛋白质,阐述蛋白质-药物相互作用,在药物靶点筛选领域大显身手。常用的先进行标记再结合亲和富集的药物靶点表征方法需要对药物分子化学衍生,这既耗时也可能会改变药物的亲和力,同时衍生基团产生的空间效应可能会阻断药物和靶点相互作用,加大了靶点鉴定的难度。因此,无需衍生的药物靶点鉴定方法受到广泛关注。蛋白质受到温度、pH、变性剂以及机械力等条件的影响会发生变性、展开和沉淀。小分子药物与蛋白质结合可能改变蛋白质折叠平衡,稳定靶蛋白的构象,与游离蛋白相比,更能抵抗不同条件诱导的蛋白质沉淀。基于以上原理,联合蛋白质组学和生物质谱技术,目前已经发展了多种基于蛋白质沉淀的药物靶点规模化鉴定方法,包括热蛋白质组分析方法、溶剂诱导蛋白质沉淀方法、机械应力诱导蛋白质沉淀方法、pH依赖性蛋白质沉淀方法等,用于蛋白质组范围内的药物靶点筛选。本综述介绍了该领域近十年报道的药物靶点发现和药物-蛋白质相互作用研究方法,总结了这些方法的特点及其在药物疗效评价、药物发现等方面的发展前景。

## Abstract (en)

Drug targets are biological macromolecules that bind drug molecules in vivo. Therefore, the system-wide identification of drug targets plays a vital role in fully understanding the mechanism of drug action, efficacy, and side effects. The unbiased screening of drug targets may accelerate the process of drug discovery and candidate screening. Mass spectrometry is a key tool for large-scale protein identification and accurate quantification owing to its high acquisition speed, resolution, and sensitivity. Mass spectrometry-based proteomics has been widely used for drug-target screening. It can systematically identify the protein-target landscape of a drug and elucidate drug-protein interactions. Commonly used drug-target characterization methods, such as labeling-based affinity enrichment, require the chemical derivatization of drug molecules, which is not only time-consuming but may also affect the affinity of the drug towards its targets. Furthermore, the spatial effects of the derivatization groups may block interactions between the drug and its targets. Considering the disadvantages of affinity-enrichment methods, strategies that do not require chemical derivatization have received widespread attention. Proteins may undergo denaturation, unfolding, and precipitation under different conditions such as high temperatures, extreme pH, denaturants, and mechanical stress. Binding to small-molecule drugs may alter the folding balance of target proteins. The conformational stability of target proteins can be stabilized by binding with drugs, and protein-drug complexes are more resistant than free proteins to the precipitation induced by different conditions. Based on this mechanism, various large-scale drug-target identification methods using protein precipitation have been developed by combining proteomics and mass spectrometry analysis, including thermal proteome profiling and solvent-, mechanical stress-, and pH-induced protein precipitation. These methods have been successfully applied to the characterization of small-molecule drug targets. In this review, we describe the protein precipitation-based methods used for the high-throughput discovery of drug targets and elucidation of the interactions between drugs and proteins in the past decade. We also summarize the characteristics of each method and discuss their application potential in drug-efficacy evaluation and drug discovery.

大多数临床可用药物通过结合一种或几种靶蛋白发挥治疗效果,药物疗效很大程度上取决于药物与靶点结合的亲和力,而不良反应通常是由于药物在毒性易感细胞中的过度结合或与其他蛋白质的脱靶结合所致。药物靶点的识别在医学指导、患者分层策略、不良反应预测以及促进临床应用等方面发挥着重要作用^[1]^,因此系统性识别药物靶点是药物全面开发的关键一步^[2,3]^。随着生物质谱技术的发展,基于质谱的蛋白质组学可以在短时间内实现几乎完整的蛋白质组分析^[4]^,为阐明疾病失调机制和监测药物作用提供了独特的功能维度信息^[5,6]^,在药物靶点全面系统鉴定中发挥着重要作用^[7]^。

早期药物靶标的鉴定是基于亲和富集的方法,首先将药物分子进行化学衍生以修饰亲和标签(一般为生物正交基团或富集基团),与细胞裂解液孵育后通过亲和反应基团分离出与药物分子相互作用的蛋白质。早期应用此方法发现环状四肽trapoxin在哺乳动物中的作用靶点是组蛋白去乙酰化酶(HDACs)^[8]^;鉴定出FKBP12是免疫抑制剂大环内酯FK506和雷帕霉素(rapamycin)的受体^[9]^。随着以质谱为基础的蛋白质组学的飞速发展,亲和富集方法已被用于系统地探索药物的蛋白质靶点,为全面分析蛋白质组内的药物相互作用提供了一种灵敏且特异的工具。例如,Ong等^[10]^发展了一种将亲和富集与定量蛋白质组学相结合的方法,为研究小分子药物作用机制提供了强有力的工具,成功应用于激酶抑制剂的靶点鉴定^[11]^。然而由于蛋白质组具有高动态范围的特点,分析结果偏向于高丰度的蛋白质,低丰度的靶蛋白可能会被遗漏,导致假阴性结果。因此,这种方法一般适用于研究丰度较高的蛋白质药物。此外,为实现亲和富集而在药物上引入的衍生基团可能会改变小分子的特异性或亲和力,从而导致对靶标的假阳性识别;同时衍生基团产生空间效应,也增加了靶点确定的难度。因此在基于亲和富集的方法中保持化学衍生药物的结构和活性是具有挑战性的。

考虑到亲和富集的不足,迫切需要发展无需药物衍生的高灵敏度方法,以期实现蛋白质组范围内的药物靶点指认。众所周知,药物与天然状态下的蛋白质结合可以改变蛋白质的折叠平衡,药物-蛋白质复合物的能态比游离蛋白质低,因此与游离蛋白质相比,药物结合后的蛋白质需克服更高的能量势垒方能达到展开状态^[12]^,从而稳定蛋白质的构象。蛋白质在不同条件下会发生变性、展开和沉淀,包括高温、极端pH值、氧化剂、有机溶剂及机械应力(图1)。从理论上讲,结合了药物的蛋白质比游离蛋白质更稳定,可以抵抗不同条件诱导的沉淀,因此这些促使蛋白质沉淀的方法都可以为药物靶点鉴定提供思路。该思路无需对感兴趣的药物进行化学衍生化,因此受到研究者们的青睐。研究者们以此为基础,开发了多种基于蛋白质沉淀的靶点筛选方法用于破译药物-蛋白质相互作用,本文归纳总结了近十年来报道的相关新方法并进行评述。

**图1:**
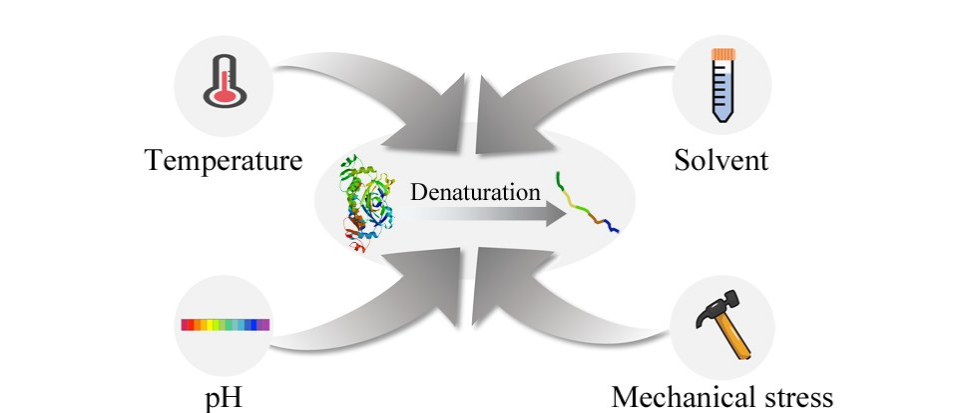
诱导蛋白质变性的几种条件

## 1 基于热诱导沉淀的方法

### 1.1 可溶性组分的热稳定性变化分析

2013年,*Science*期刊上报道了一种直接监测细胞内药物与蛋白质相互作用的方法,称为细胞热迁移实验(cellular thermal shift assay, CETSA)方法^[13]^。其原理是当蛋白质受到热刺激时,通常不可逆地展开并暴露出疏水核心,进而蛋白质聚集生成沉淀。在同等温度下,与药物结合后的蛋白质更加稳定,抵抗高温诱导的展开,从而抵抗热诱导沉淀,使得热熔曲线发生明显变化。因此可通过比较药物及载体对照处理的样品在高温下可溶性蛋白质的含量来测定蛋白质稳定性的变化,以验证药物结合的目标蛋白质。具体而言,等份的细胞裂解液经过一系列升温处理,冷却后离心得到可溶性部分,通过蛋白质印迹(Western blot)对可溶性馏分中的目标蛋白质定量,进而绘制热熔曲线。应用此方法验证了一系列与重要蛋白靶点结合的药物,监测了药物在癌细胞系中的转运、激活。此外,此方法也用于动物组织裂解液中药物靶点验证及药物分布研究。CETSA方法是验证药物靶点结合的有价值的工具。

得益于以质谱为基础的蛋白质组学的飞速发展,Savitski及其同事^[14,15]^进一步扩展了CETSA方法以突破药物发现的瓶颈,即鉴定药物靶点、脱靶点以及挖掘响应药物功效的分子生物标志物。他们将CETSA方法与基于多重定量质谱的蛋白质组学相结合,建立了细胞中全蛋白质组范围的热稳定性测定方法,称为热蛋白质组分析(thermal proteome profiling, TPP)方法。此方法可同时监测10种温度条件下可溶性蛋白质的热稳定性变化,通量绘制多种蛋白质的熔化曲线。与CETSA方法类似,用药物和载体对照处理细胞后,将样品分成10等份,每等份短暂加热到相应温度,同样收集可溶性馏分,每个温度点样品经胰蛋白酶处理后用不同的稳定同位素质量标签标记,10种温度条件下的样品混合后通过LC-MS/MS分析,利用MS/MS谱图中获得的肽段报告离子强度绘制可溶性蛋白馏分的完整熔化曲线并计算每种蛋白质的熔点。不加药物处理的细胞也进行相同操作,最后通过药物结合引起的熔点变化来确定目标蛋白质,以此得到药物靶点等感兴趣的信息。为了验证TPP方法识别结合靶标的能力,利用TPP方法监测了广谱型抑制剂星形孢菌素(staurosporine)和GSK3182571的结合靶点,发现多种激酶靶点的熔化温度发生变化,并影响包括激酶复合物的调节亚基在内的其他蛋白质热位移;此外他们发现亚铁螯合酶FECH是几种激酶抑制剂的脱靶点。TPP方法一经报道备受科学家关注,其不仅在揭示药物靶标和非靶标方面取得了巨大成功,在探测代谢物-蛋白质相互作用^[16]^、蛋白质-蛋白质相互作用^[17]^,甚至是蛋白质翻译后修饰方面也取得了巨大成功^[18]^。

CETSA方法也应用于检测体内给药后组织中单个药物靶点的热稳定性变化^[19]^; TPP方法在细胞和裂解液层面规模化监测蛋白质的热稳定变化,实现了蛋白质组范围内药物-蛋白质相互作用的研究;然而为了更直观准确地预测药物疗效和不良反应,将靶标和脱靶标鉴定从简单的细胞体系转化为体内研究充满了挑战^[20]^。直到2020年Perrin等^[21]^基于TPP方法,发展了组织TPP(tissue thermal proteome profiling, tissue-TPP)方法,其通过使用定量质谱法检测蛋白质组热稳定性变化来评估药物治疗动物组织样品中小分子药物与蛋白质的结合以及可能的脱靶效应,帮助阐明药物在生物体内发挥作用的机制。通过静脉注射非共价组蛋白去乙酰化酶抑制剂帕比司他(panobinostat)进入大鼠体内,取组织切片,按照TPP方法进行定量质谱分析,与在体外直接帕比司他处理大鼠组织切片相比,蛋白质组的热稳定性变化具有较高的一致性,同时绘制和概括了帕比司他在大鼠肝脏、肺、肾脏和脾脏中的热稳定性图谱和靶点。此外,相较于动物组织活检,在人体研究中通常更容易获得血液样本,因此研究者们将tissue-TPP方法扩展优化以应用于全血样品中热蛋白质组稳定性分析(称为blood-TPP)方法,血液样品与帕比司他孵育后,无需额外的实验步骤即可直接进行热处理,最终结果揭示了HDAC1、HDAC2、HDAC6靶点以及TTC38脱靶点,与在细胞系和组织切片中的结果一致,还发现了新的靶蛋白ZNF512,表明了blood-TPP方法在临床环境中应用的潜力。

CETSA方法可以监测活细胞中药物处理后蛋白质的热稳定性变化,核实药物的目标靶蛋白,实现生理相关环境中药物-蛋白质相互作用的评估,这是药物发现研究的重要一步,但是无法发现其他潜在的靶点。因为大多数候选药物甚至批准的药物可能有多个生理靶点,为此TPP方法应运而生,其可以并行评估数千种蛋白质以获得直接和间接的药物靶点^[15]^,提前发现药物与意想不到的靶点相互作用所介导的毒性作用以及缺乏靶点参与造成的药物疗效降低^[22]^,为全面理解和合理设计药物提供了数据参考。值得一提的是,这些方法仅分析可溶性组分,遗漏了含有丰富药物稳定性变化信息的沉淀组分。

### 1.2 沉淀组分的热稳定性变化分析

分析热诱导后蛋白质组样品的可溶部分,这一策略已在药物靶标筛选领域大显身手。理论上,上清液与沉淀组分均可揭示药物结合的靶蛋白热稳定性变化;然而由于需要再溶解沉淀组分中的蛋白质,会影响批次重复性,同时有限的样品产生的沉淀较少甚至看不见,难以处理,这些原因使得沉淀组分的研究更为困难,阻碍了其应用和发展。得益于在蛋白质组样品制备中大展拳脚的微粒辅助的蛋白质聚集捕获(microparticle-based protein aggregation capture, PAC)方法的出现^[23]^, Lyu等^[24]^在TPP方法与PAC方法的基础上提出了微粒辅助沉淀筛选(microparticle-assisted precipitation screening, MAPS)方法。在药物或载体处理的细胞裂解液中加入磁性微粒(羧基修饰,粒径1 μm),经过热处理后磁性分离得到微粒,最后将沉淀在微粒上的蛋白质进行洗涤、烷基化、酶解、标记和定量蛋白质组分析。热诱导的蛋白质沉淀过程由微粒辅助,加热后蛋白质展开并聚集在微粒表面,与游离蛋白质相比,与药物结合的靶蛋白更能抵抗微粒表面的聚集;基于此原理,药物处理组和载体处理组在微粒表面聚集的热稳定性差异蛋白质即为药物靶蛋白。使用MAPS方法成功鉴定到甲氨蝶呤(methotrexate)的直接作用靶点二氢叶酸还原酶(DHFR),同样地也找到了雷替曲塞(raltitrexed)、环孢菌素A(cyclosporin A)和SHP099的已知靶点,证明了MAPS方法筛选药物靶点的可行性;靶向格尔德霉素(geldanamycin)的蛋白质和脱靶蛋白质也被找到;此外,在星形孢菌素的靶点鉴定中,MAPS方法共找到32种蛋白激酶,特异性高达80%。

微粒的引入简化了整个样品制备工作流程,整个过程在微粒表面方便地进行,无需任何转移,使得样品损失最小化。值得一提的是,MAPS方法的初始蛋白质使用量仅20 μg,是TPP方法的1/10,因此MAPS方法与微量样本中药物靶点研究相兼容的优势填补了针对沉淀组分的靶标筛选策略的空白。然而利用细胞裂解物研究药物靶点,MAPS方法只能揭示与药物分子相互作用的直接靶点,而无法识别与药物代谢物相互作用的间接靶点;此外因需要使用微粒辅助,此方法无法用于活细胞中的研究。

基于热诱导的蛋白质稳定性变化,可溶性组分与沉淀组分含有互补信息,将两者结合起来可以提高药物靶点识别的特异性和灵敏度。Ruan等^[25]^将热诱导的沉淀和上清液含有的蛋白质稳定信息结合,联合内部开发的基于深度学习的数据处理算法IBR-CNN,发展了包括沉淀组分信息的TPP(precipitate-supported TPP, PSTPP)新方法,实现更加全面的药物靶点信息指认。该研究使用模型药物星形孢菌素评估PSTPP方法的性能,确定了99个激酶靶点,灵敏度提高了94%。基于PSTPP方法良好的性能,研究者进一步表征了雷帕霉素的结合靶点;结果显示已知靶点FKBP1A在鉴定中表现出最明显的稳定性,同时KFBP2和KFBP3也有明显的稳定性。尽管FKBP1A有显著的稳定性,但其仅在沉淀组分中观察到明显位移,再次突出了同时检测沉淀组分的重要性。此外,PSTPP方法仅使用更有效的温度范围(47~59 ℃),与TPP方法相比,显著简化了实验流程,缩短了大约一半的时间及串联质量标记(tandem mass tag, TMT)试剂。与MAPS方法不同的是,此方法可用于活细胞中药物靶点的研究。

总之,热稳定性相关方法(图2)广泛适用于基础研究以及临床前和临床环境。需要注意地是,并非所有的蛋白质都容易沉淀,特别是在与细胞结构完整性相容的温度下,因此,对于小的和亲水的蛋白质,展开后的聚集可能是不完整的,或者根本不发生。因为膜蛋白不易溶解,最初的方法并不适用于靶向膜蛋白的药物研究,经过研究者不懈努力,此方法可以扩展应用于跨膜蛋白-药物相互作用的研究^[26⇓-28]^。然而该方法无法研究对温度没有反应的靶蛋白,同时熔化曲线拟合不良的蛋白质会被过滤掉,这也增加了丢失重要靶点或功能蛋白的风险。除此之外,TPP方法需要在10个温度下加热大量样品以生成完整熔化曲线,昂贵的TMT试剂以及复杂繁琐的工作流程也阻碍了TPP方法的进一步应用。

**图2:**
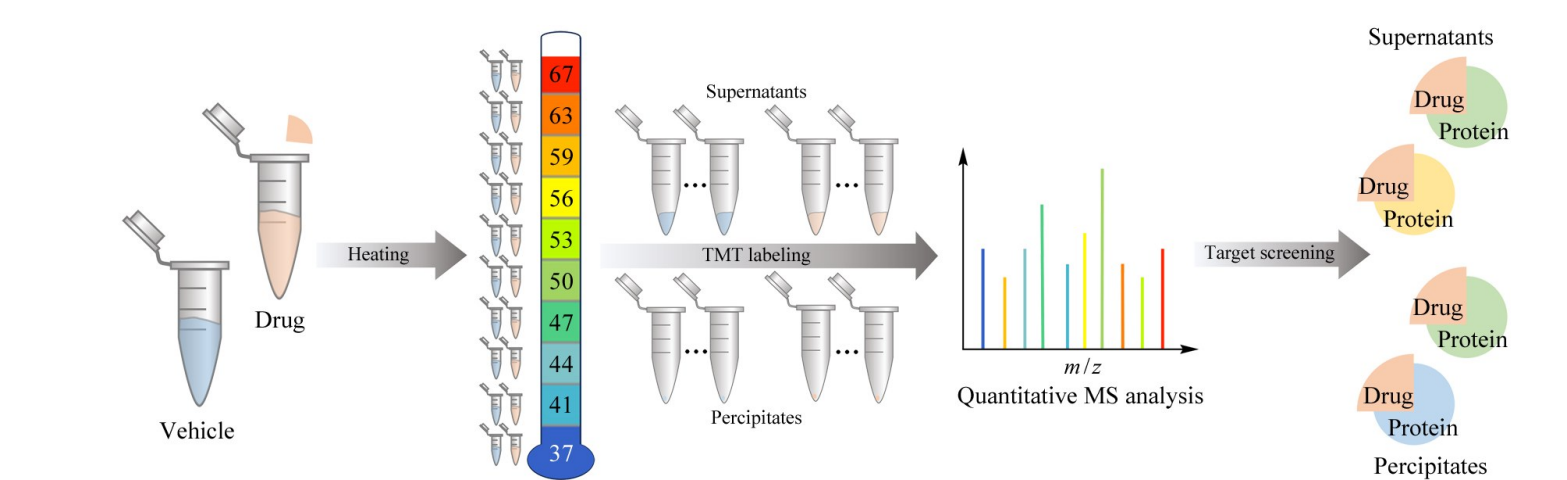
基于热诱导方法药物靶点鉴定流程图(仅展示重要步骤)

## 2 溶剂诱导蛋白质沉淀的方法

有机溶剂通过降低溶液介电常数和破坏蛋白质水化膜使蛋白质变性和沉淀^[29]^;药物-蛋白复合物具有较低的能态,与游离蛋白相比需要更多的能量来展开,因此,与药物结合的靶蛋白对有机溶剂诱导的变性和沉淀具有更强的抵抗力。Zhang等^[30]^利用这一特点发展了溶剂诱导蛋白沉淀(solvent-induced protein precipitation, SIP)方法来确定药物的靶点以及脱靶效应(图3)。该方法使用实验室常用的化学试剂,如丙酮、乙醇、乙酸等。两等份的细胞裂解液分别与药物和对照载体孵育后,添加有机溶剂混合物(丙酮-乙醇-乙酸,体积比为50∶50∶0.1)引发蛋白质变性和沉淀,离心得到的可溶性蛋白质经蛋白质组学样本前处理和二甲基化标记后进行质谱鉴定,定量蛋白质组分析确定药物的靶点。使用已知靶点的模型药物甲氨蝶呤和SNS-032经SIP方法处理后质谱分析鉴定出了甲氨蝶呤的靶点DHFR,同样地也找到了SNS-032的两个已知靶点CDK2和GSK-3α,因此表明了SIP方法在药物特异性靶点指认方面的可行性。进一步使用泛激酶抑制剂星形孢菌素验证了SIP方法识别靶标结合的能力,尽管质谱鉴定到了上千种蛋白质,但仅有很少量的蛋白质与药物结合后发生明显变化,经过定量蛋白质比较筛选出9种激酶靶点,同时还发现了11种与药物结合后稳定的非激酶,这与之前报道的非激酶可能是激酶抑制剂的靶点的结论相一致^[14]^。格尔德霉素因严重的副作用已退出临床^[31]^,应用SIP成功鉴定出其3个已知蛋白靶点HSP90AA1、HSP90AB1和HSP90B1,并首次鉴定出NADH脱氢酶亚基NDUFV1和NDUFAB1等多个潜在脱靶蛋白,这可能是造成副作用的原因^[30]^。Meng等^[32]^利用化学变性剂诱导蛋白质沉淀的方法评估药物诱导的蛋白质折叠自由能变化,以识别药物的蛋白质靶点,结合基于质谱的蛋白质组学方法,规模化量化蛋白质-药物的相互作用,称为化学变性剂诱导蛋白沉淀方法(chemical denaturant and protein precipitation, CPP)。CPP方法与SIP方法流程类似,不同的是使用盐酸胍(GdmCl)代替有机溶剂使蛋白质变性沉淀。CPP方法识别了环孢菌素A和格尔德霉素的已知靶点,证明了方法的可行性;同时应用于泛甲基转移酶抑制剂西奈芬净(sinefungin)的靶点鉴定。

**图3:**
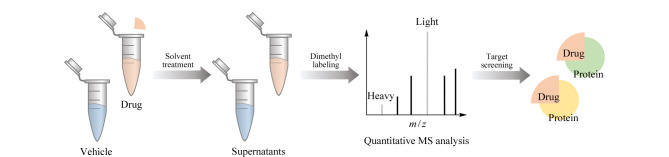
SIP的流程图(仅展示重要步骤)

总而言之,诱导蛋白质聚集沉淀的有机溶剂和化学变性剂均是实验室常用的试剂,方法简单;对热不敏感的药物靶点可能对溶剂诱导敏感,意味着不同方法识别的靶点信息可以相互补充,为药物靶点识别提供了强大平台,以更好地了解药物的副作用和作用机制。然而SIP方法仅分析可溶性部分,含有丰富药靶证据的沉淀组分被舍弃,可能会遗漏部分重要信息。

## 3 机械应力诱导的方法

蛋白质通过混合和搅拌承受很高的机械应力,导致蛋白质聚集甚至沉淀^[33]^。基于此,机械应力可以作为药物靶点筛选的潜在驱动力;蛋白质在机械应力作用下会发生变性,导致蛋白质聚集,而结合药物的蛋白质复合物会阻止靶蛋白的聚集。然而,在运输过程中,蛋白质产品总是经过长时间的振荡,然后开始沉淀。也就是说,机械应力一般需要较长时间才能诱导蛋白质沉淀,这不利于实验室靶点筛选,因此Lyu等^[34]^在蛋白质溶液中引入微粒形成涡流来提高机械应力;在汽液、固液和液液界面处的界面应力可诱导和强化蛋白质聚集^[35]^,以此加速蛋白沉淀,缩短时间,由此发展了机械应力诱导蛋白质沉淀(mechanical stress induced protein precipitation, MSIPP)方法(图4),扩充了无修饰药物靶点筛选方法。与前面介绍的MAPS方法类似,将诱导蛋白质变性的机制由加热替换为机械应力。药物和载体对照处理的细胞裂解液加入微粒后置于涡旋混匀器上涡旋,以诱导蛋白质聚集沉淀于微粒表面,磁性分离后进行蛋白质组学分析。由于药物结合蛋白质对机械应力的抵抗力更强,药物处理组的蛋白质沉淀会比载体处理组少。使用MSIPP方法成功地鉴定出4种临床药物(雷替曲塞、甲氨蝶呤、SHP099和格尔德霉素)的已知靶点以及星形孢菌素的几十种靶点;此外,MSIPP方法不仅鉴定到雷替曲塞的已知靶点TYMS,还首次发现其可以与DHFR结合并影响DHFR的机械稳定性^[34]^。

**图4:**
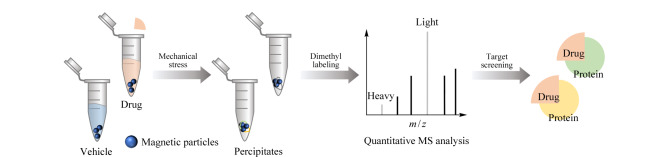
MSIPP方法的实验流程图(仅展示重要步骤)

MSIPP方法从新角度揭示药物-蛋白质相互作用,为蛋白质组水平的药物靶点筛选提供了一个标准的操作方案,是其他无修饰药物靶点筛选方法的补充。然而MSIPP方法也有自身的不足,首先微粒的引入限制了其在活细胞中的应用;其次有些蛋白质易被微粒捕获,而有些蛋白质则是天然顽固的,需要更多的机械应力才能沉淀。因此当使用不同的机器提供机械应力时应优化机械应力诱导沉淀的参数。

## 4 pH诱导的方法

除了前面提到的蛋白质变性方法,改变pH值也可以使蛋白质变性。降低pH会使蛋白质的氢键断裂并带正电,从而与酸性试剂阴离子形成不溶的络合物^[36,37]^。基于此原理,Zhang等^[38]^报道了pH依赖性蛋白质沉淀(pH-dependent protein precipitation, pHDPP)方法应用于蛋白质组规模的药物靶标鉴定(图5)。具体思路如下:在药物及载体对照处理的细胞裂解液中加入酸性试剂(抗坏血酸、柠檬酸、甲酸、盐酸)以诱导蛋白质变性沉淀,离心取上清液进行蛋白质稳定性分析。使用抗坏血酸研究甲氨蝶呤的靶点,与预期一致,结合了甲氨蝶呤的DHFR在抗坏血酸诱导中有显著的稳定性;使用基于柠檬酸的pHDPP方法不仅鉴定到甲氨蝶呤的靶点DHFR,还发现了另一个靶点TYMS。以柠檬酸诱导环孢菌素A处理的HeLa细胞裂解液,收集上清液和沉淀分别进行分析,结果表明在上清液和沉淀中均鉴定到了靶蛋白PPIA,这说明pHDPP方法可以同时分析上清液和沉淀组分来双重定位药物靶蛋白。有趣的是,PPIA在沉淀中显示出更好的特异性,这提示对于低丰度的蛋白质,沉淀物中蛋白质丰度变化的检测可能更加灵敏和准确。使用星形孢菌素处理的K562细胞裂解液比较了pHDPP方法、TPP方法和SIP方法,3种方法鉴定到了数目相似的潜在靶点,进一步的靶点分析发现,pHDPP方法检测到42个蛋白激酶,而TPP方法和SIP方法仅检测到36个和34个蛋白激酶,同样pHDPP方法中非蛋白激酶的百分比(14%)明显低于内部TPP方法(29%)和SIP方法(31%),表明酸性试剂处理比加热和溶剂处理更能使这些蛋白激酶产生差异沉淀。3种方法共鉴定到67个蛋白激酶,作为广谱激酶抑制剂,这67种靶激酶分布在不同的激酶家族中;因每种方法都有特异性识别的蛋白激酶,整合3种方法可以提高药物靶点鉴定的覆盖率,数据表明,3种方法在靶点识别方面具有很好的互补性。最后应用pHDPP方法,在双氢青蒿素(DHA)中筛选出了45个潜在靶点;接着通过分子对接技术和基于人工智能靶标预测的MolDesigner软件包评估了这些潜在靶点,发现ALDH7A1和HMGB1两个靶点在两种评估中表现良好,进一步通过CETSA方法验证了ALDH7A1和HMGB1与DHA具有较强的亲和力,表明pHDPP方法在药物靶点鉴定中具有高可信度。

**图5:**
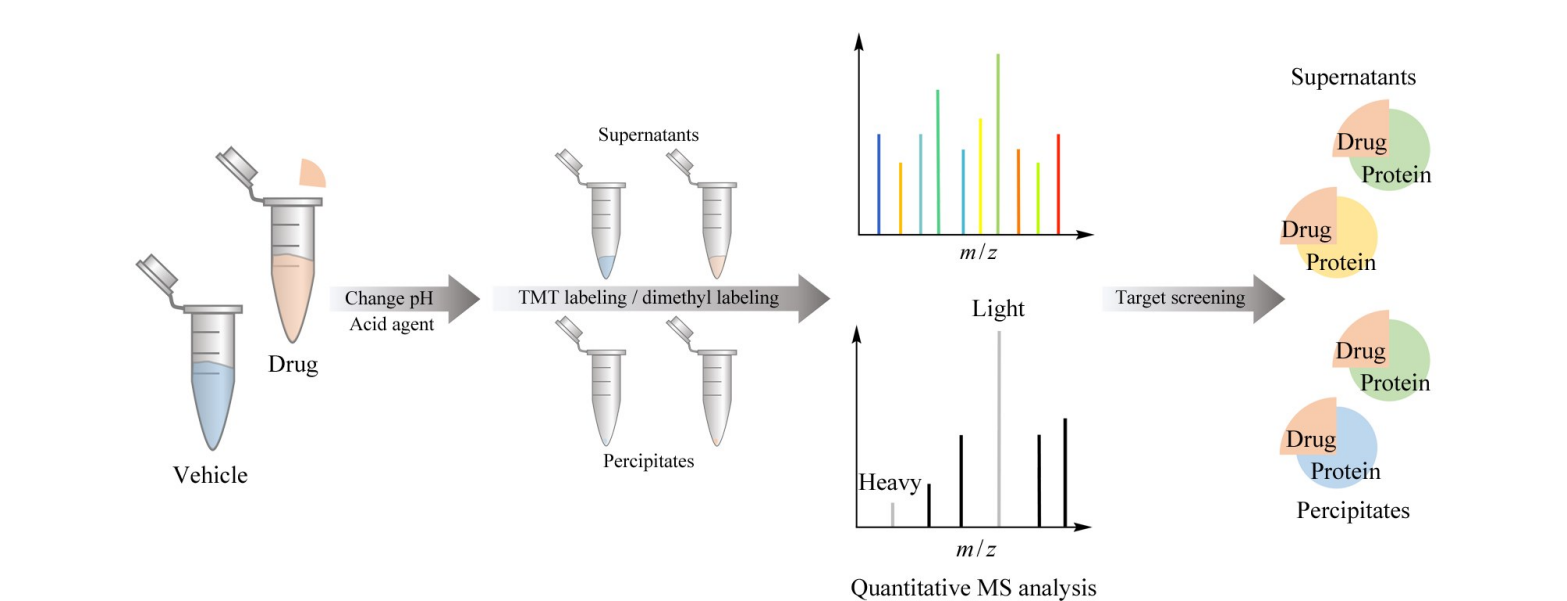
pH依赖性蛋白质沉淀方法的流程图(仅展示重要步骤)

总之,pHDPP方法是鉴定药物靶蛋白及揭示药物作用机制的有力工具。它可以实现蛋白质组范围内的靶标识别,而不需要化学修饰。工作流程简单,节省时间。然而,酸性试剂的使用可能会改变一些小分子药物的酸碱平衡,从而调节它们与靶蛋白的相互作用,需要更多的实验来验证药物靶点发现的准确性。

综上,4种基于蛋白质沉淀的药物靶点筛选方法共有优点是无需进行额外的化学修饰,当然每种方法有相应的优缺点和适用范围(表1),因此在药靶筛选中可以用多种方法同时鉴定,鉴定靶点相互补充,以实现更加全面的靶点筛选。

**表1:** 4种基于蛋白质沉淀的药物靶点筛选方法的优缺点和适用范围

Method	Advantages	Disadvantages		Applications	
Temperature- induced	compatible with multiple types of samples, reflecting the natural binding of proteins and drugs		unable to identify targets that are unre- sponsive to temperature changes		cell lysis, living cells, tissues, blood
Solvent-induced	common reagents, simple procedures		can only analyze soluble fractions		cell lysis
Mechanical stress-induced	compatible with mild detergents, simple workflow by microparticle assistance		cannot be applied to living cells		cell lysis
pH-induced	common reagents, low cost, simple workflow		shifting acid-base equilibrium for some drugs		cell lysis

## 5 总结与展望

因为亲和富集方法的靶点鉴定有偏向性,因此需要设计合成多种衍生基团修饰的药物来全面表征药物靶点,这是非常耗时耗力的。相反,基于蛋白质沉淀的直接靶标分析可以在广泛的亲和力范围内检测小分子-靶点相互作用,不需要耗时的药物衍生设计,同时借助质谱蛋白质组技术高灵敏和深覆盖的特点使该策略更具吸引力。本文从诱导蛋白质沉淀的角度出发,综述了近十年来药物靶点鉴定方法的发展,这些方法的显著特点是不需要对药物进行修饰,在药物靶点鉴定和发现方面取得了显著进步。值得注意的是,由于在不同变性条件下蛋白质的敏感性不同,使得蛋白质溶解度不同,利用不同变性条件沉淀蛋白质的方法进行药物靶点筛选时,可能会鉴定出不同的靶点。同时与经典的亲和富集方法相比,无衍生化方法的靶点发现能力仍有不足,研究发现将泛抑制剂星形孢菌素化学衍生后亲和富集,在K562细胞中发现229个靶点^[39]^,而使用无修饰的方法(TPP方法)在相同细胞系仅鉴定到51个蛋白激酶^[14]^。因此在药物靶点鉴定时,需要结合不同方法,获得互补信息,实现更全面的靶点鉴定。每种方法都有优点和局限性,但各种成功案例证明了无需药物衍生的药靶筛选方法在识别疾病和药物作用机制、指认脱靶作用以及提供药物再利用证据等方面的可行性。将这些方法系统地嵌入到药物发现进程中可以极大地缩短药物研发周期,及时止损,减少后期损耗。

基于质谱的蛋白质组技术在阐明疾病失调机制和监测药物作用研究中发挥着重要作用,可用的仪器、软件和工作流程变得越来越成熟和强大。尽管与RNA测序等其他组学技术相比,蛋白质组学在灵敏度和覆盖度方面仍有不足,但它可以提供与疾病和药物作用机制直接相关的信息。目前各种自动化样品制备平台层出不穷,质谱技术和机器学习飞速发展以及相关方法在微量样本中的应用,使得未来在少数或单个细胞中同时实现快速且高通量的多种药物靶点筛选和作用机制研究成为可能。

## 作者团队简介

蛋白质分离分析新技术课题组隶属于北京蛋白质组研究中心,在课题组长的带领下,发展了一系列特色性的低丰度/修饰蛋白质和外泌体富集材料,核酸-蛋白复合物、血液和单细胞蛋白质组分析新方法,显著提高了鉴定灵敏度和规模。特别是围绕作为重要诊断标志物和药物靶点的糖基化蛋白质,建立了多种高效富集、质谱鉴定和数据解析新方法,为糖蛋白质组研究提供了系统性工具。先后主持国家重大科学研究计划课题、国家重大科学仪器设备开发专项课题、国家重点研发专项课题以及国家自然科学基金面上项目等。

### 人才队伍

**Figure:**
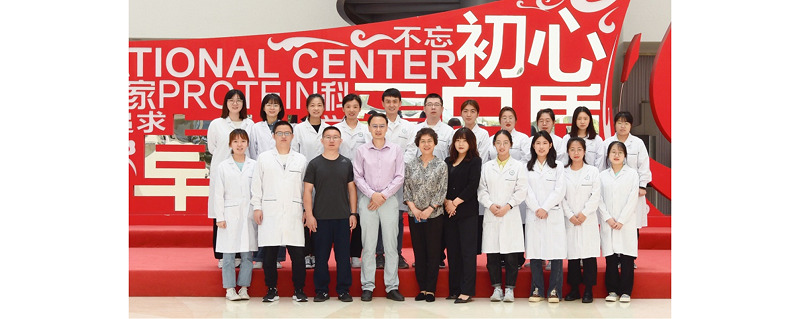


**课题组组长:**秦伟捷研究员

**职工及学生:**研究员1人,副研究员1人,博士后及研究生20余人

**团队精神:**追求真理,勇于探索,唯实唯真,潜心研究

### 科研项目及成果

**科研项目:**国家重大科学研究计划课题,国家重大科学仪器设备开发专项课题,国家重点研发专项课题以及国家自然科学基金面上项目等

**科研成果:**在*JACS*, *Angew*, *Molecular Cell*, *Nature Chemical Biology*, *Chemical Science*, *Analytical Chemistry*, *Nucleic Acids Research*等国际知名期刊发表论文80余篇。

**获奖情况:**国家科技进步二等奖等

**Figure:**
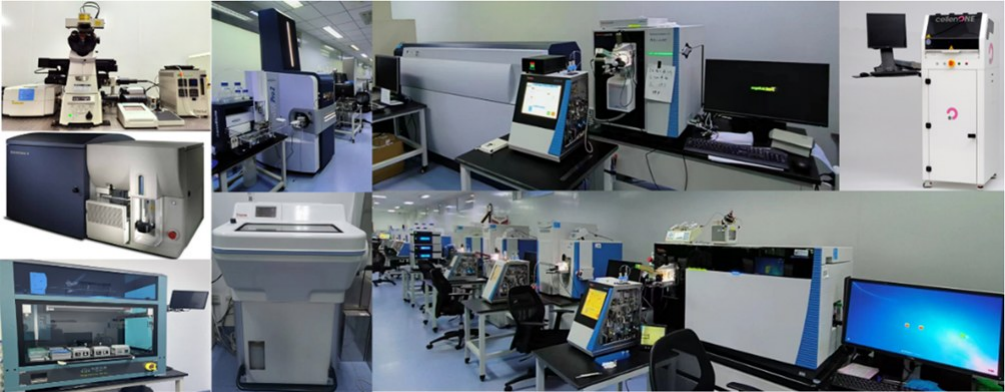


### 研究领域

**Figure:**
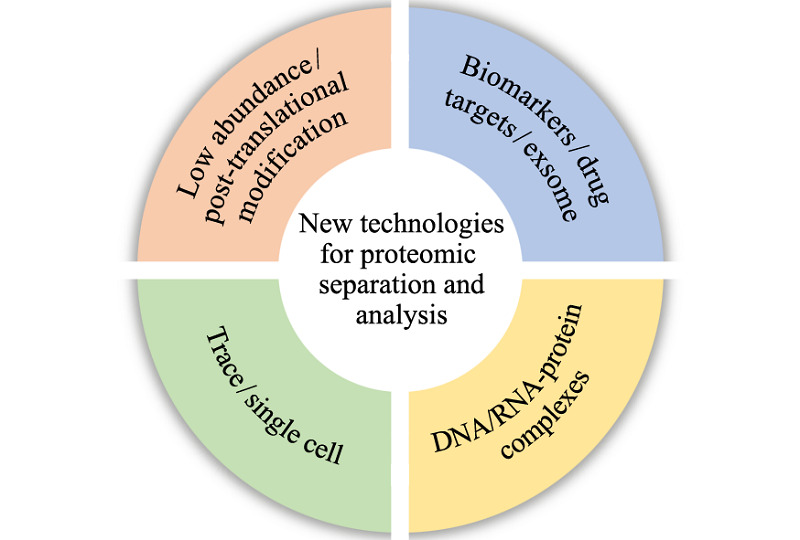


### 仪器设备信息

TIMS-TOF-Pro2高分辨质谱仪,FAIMS Orbitrap Exploris 480高分辨质谱仪,VION IMS QTOF液相色谱-质谱联用系统,Orbitrap Fusion Lumos三合一高分辨质谱仪,Q Exactive HF高分辨质谱仪,全自动单细胞分选仪,冷冻切片机,流式细胞分选仪,超高分辨率共聚焦显微镜成像系统,蛋白质组学全流程自动化前处理机器人,生物分子成像仪等
